# The Friction-Induced Vibration of Water-Lubricated Rubber Bearings during the Shutdown Process

**DOI:** 10.3390/ma13245818

**Published:** 2020-12-20

**Authors:** Guangwu Zhou, Peng Li, Daxin Liao, Yuhao Zhang, Ping Zhong

**Affiliations:** 1 School of Aeronautics and Astronautics, Sichuan University, Chengdu 610065, China; lipeng@stu.scu.edu.cn (P.L.); liaodaxin@stu.scu.edu.cn (D.L.); 2019226210006@stu.scu.edu.cn (Y.Z.); 2 Industrial Turbine Division, Dongfang Turbine Co., Ltd., Deyang 618000, China; zhongping@dongfang.com

**Keywords:** tribology, friction-induced vibration, water-lubricated rubber bearings, shutdown process, transient dynamics

## Abstract

The vibration noise generated by water-lubricated rubber bearings (WLRBs) seriously reduces the concealment of a ship’s navigation. The purpose of this study was to obtain the relationships between friction-induced vibration and the friction coefficient, specific pressure, temperature, and stiffness of the bearing support during the shutdown process of WLRBs. Using transient dynamic analysis (Abaqus/Standard), the shutdown process of the bearing system was simulated by setting a friction coefficient curve, and with the fast Fourier transform (FFT), the data in the time domain were then converted to the frequency domain. In addition, an orthogonal table was applied to select the best level for each factor. The results show that proportionally increasing the friction coefficient and specific pressure caused higher vibrations, and the effect of the specific pressure on vibration is more prominent than that of the friction coefficient. Higher temperatures led to an increase in the peak frequency of noise (squeal) and the virtual value of acceleration. Increasing the stiffness of the bearing support decreased the higher-frequency squeal but dramatically increased the lower-frequency chatter. The results of the study are of guiding significance for the improvement of research methods and the optimization of the materials and structures of WLRBs.

## 1. Introduction

**Figure 1:**
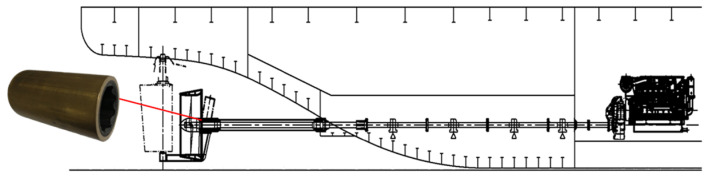
Marine propulsion shafting and stern bearing.

A water-lubricated rubber bearing (WLRB) is an important part of the stern tube system in marine propulsion shafting. It supports the stern shaft and is an important part of the internal and external power exchange. A sketch of the marine propulsion shafting is shown as Figure 1. Unlike the traditional oil-lubricated sliding bearings, water-lubricated bearings, with the advantage of high energy efficiency, long life, no pollution, and so on, are resource-saving and environment-friendly bearings. However, it has been shown that the thickness of a water film is only about one-eighth of the thickness of an oil film under the same carrying capacity, which indicates that the carrying capacity of a water-lubricated bearing is relatively low and waterless lubrication and dry friction are very likely to occur. Because rubber material has a strong nonlinear property and the water-lubricated bearings mounted in a marine propulsion system always operate under a low speed, a heavy load, and other complex conditions for a long time, as a result, friction vibration and noise are generated, which seriously affects the working and concealment performance. Therefore, the vibration noise of a WLRB needs to be reduced.

Bhushan [1] carried out experimental research on the noise mechanism for a WRLB and the results revealed that a stick-slip motion, a mechanism related to the change rate of the friction coefficient with velocity, is the source of the noise. Krauter [2] conducted a simulation for the friction noise of a WRLB and it was concluded that the slope of the friction–speed curve and the structural damping are the most important factors related to squeal/chatter. Simpson et al. [3] proposed a dynamic model of water-lubricated bearings with two nonlinear degrees of freedom and the results revealed that a high-frequency vibration superimposed on the fundamental frequency of a system generates squeal. Based on a nonlinear disk brake model and a subcritical Hopf bifurcation of the trivial solution, Hochlenert [4] performed a nonlinear stability analysis showing that sometimes the intensity of squeal is independent of the rotational speed. Hongling et al. [5] conducted experiments to study the relationship between friction-induced vibration and factors related to vibration, where the vibration of the specimens during the tests was recorded with a high-speed camera and machine vision technology, which sorted the importance of factors in the order of lubrication condition, contact pressure, hardness, and the thickness of the rubber. Zhou et al. [6] used complex mode analysis to study the effects of the friction coefficient, material, and structure of a WLRB on friction-induced vibration. Taking into account self-excited vibration, the gyroscopic effect, friction-induced damping, and brake pad geometry, Ghorbel et al. [7] proposed a disk brake model with a minimal two degrees of freedom to study the effects of various parameters on the model-coupling instability. Taking into consideration the effect of normal vibration by bringing the motion of the stern shaft into the normal load description of friction, Jin et al. [8] established an equation for friction-induced vibration to investigate the influence rule of normal vibration on friction-induced vibration. Although the friction coefficient–velocity curve was fitted, it was still studied at a constant velocity. By conducting theoretical analysis and an experimental study, Wang et al. [9] studied the effect of friction and wear on bearing vibration using bearing bushes made from different materials under different working conditions. A series of tests was used to study the lubrication and friction characteristics of a three-layer (polytetrafluoroethylene (PTFE)–nitrile butadiene rubber (NBR)–bronze) WLRB with lubrication grooves along the entire and upper-half perimeter [10,11]. Litwin and Dymarski [12] conducted experimental studies on water-lubricated bearings under the conditions of improper lubrication and cooling and found that a three-layer bearing with grooves only in the upper part of the bush is an optimal solution. As the load increases, the critical point at which the high-frequency self-vibration occurs from mild to severe friction and wear changes, which depends on the system’s rigidity [13]. The responses of the rubber, fixer, and shaft were recorded using a high-speed camera to study the process of coupling with torsional vibration [14]. The friction-induced vibration of a stern bearing at low speed shows the characteristics of randomness and nonlinearity. The experiment was also conducted at a constant speed [15]. Deleau et al. [16] simulated the contact and friction behavior of a rubber blade/windscreen by studying the friction behavior of the rubber/glass contact. It is a fact that the key to studying and reducing the friction-induced vibration and noise is to understand the generation of noise sources, and so far, many researchers have carried out experimental studies [17,18] and numerical simulations [19,20,21,22] and used finite element methods [23].

It can be seen that researchers have been studying the squeal/chatter of WRLBs using a variety of methods, where most of the previous studies have the use of a constant speed in common rather than a continuously changing speed. However, the noise of a bearing system mainly occurs during the start-up and shutdown processes, where the friction coefficient changes continuously and dynamically. Therefore, to fill this research blank, using the finite element method (FEM), this study changed the friction coefficient continuously according to the designed friction coefficient curve during the shutdown process to simulate the shutdown process of a WRLB and study the effects of the friction coefficient, specific pressure, temperature, and stiffness of the bearing support on the vibration noise. Therefore, the research content of this paper is of great significance for improving the research methods regarding vibration noise.

## 2. Establishing a Finite Element Model for a Water-Lubricated Bearing System

### 2.1. Dimensions of the Bearing System Model

**Figure 2:**
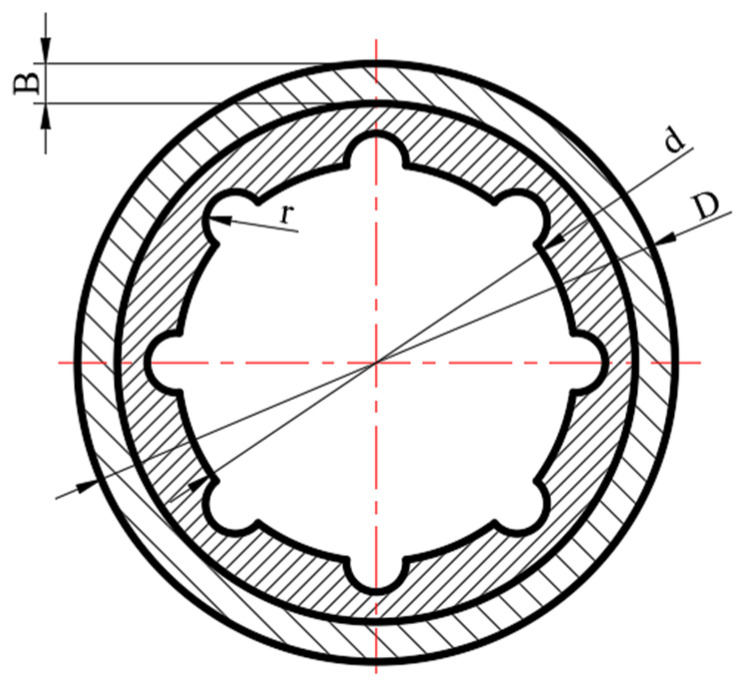
Geometric structure of the water-lubricated bearing.

**Figure 3:**
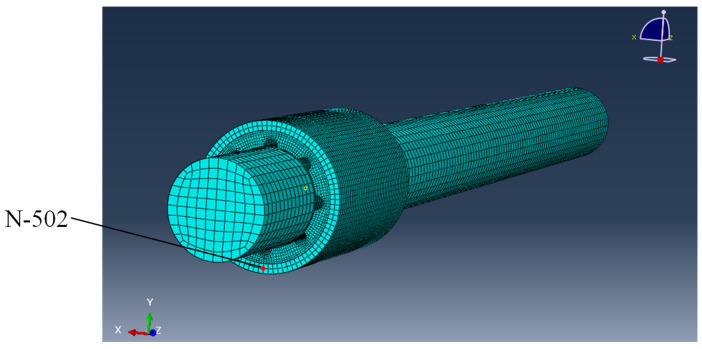
The model of the bearing system and the observation point (N-502).

A solid model was established according to the actual size of a WLRB with a specification of 40 mm × 60 mm × 80 mm. The geometric structure of the water-lubricated bearing is shown in Figure 2. The model of the bearing system is shown in Figure 3. The structure parameters are listed in Table 1.

### 2.2. Materials of the Bearing System

The bearing consisted of two parts, where the inner ring was a rubber bush and the outer ring was a brass sleeve; these were bonded together using a high-strength structural adhesive. Steel 45 in GB/T 699-2015 [24], with a Young’s modulus of 206 GPa, a density of 7800 kg·m^−3^, and a Poisson’s ratio of 0.3, was selected for the shaft. Navy brass, with a Young’s modulus of 106 GPa, a density of 8900 kg·m^−3^, and a Poisson’s ratio of 0.35, was selected for the sleeve. The rubber in this study was an NBR matrix composite, and the Mooney–Rivlin model was selected with a C_10_ of 0.794 MPa, a C_01_ of 0.139 MPa, and a density of 1500 kg·m^−3^. Rubber is typically superelastic, and NBR, with a tensile strength of 18–21 MPa, a Young’s modulus of 5.83 MPa, and a Shore hardness (A) of 63, has a better mechanical performance [25].

### 2.3. Division of the Finite Element Mesh

The division of the finite element mesh for the WLRB is shown in Figure 3, in which the highlighted N-502 (node 502) was selected as the observation point. Linear hexahedral elements of type C3D8R was applied to three parts. The number of nodes for the shaft, rubber bush, and brass sleeve was 21,131, 80,838, and 10,824, respectively, and the number of elements was 17,856, 66,240, and 7040, respectively.

### 2.4. Boundary Conditions

Whether simulation results are consistent with an actual situation depends on the rationalization of the boundary conditions. In this model, a binding constraint was adopted as the contact form between the copper sleeve and the rubber bush, and the contact property between the shaft and the bearing was defined by the surface and surface contact in Abaqus/Standard (Abaqus/CAE 2016, Dassault Systems Simulia Corp., Johnston, RI, USA). In addition, an amplitude curve was used to define the change of friction coefficient in analysis steps. The translational and rotational degrees of freedom in both the x- and y-directions at the end of the shaft away from the bearing were constrained. The external surface of the bearing was fully fixed or grounded via a spring. A concentrated force was loaded on the shaft-end face near the bearing in the negative y-direction and a continuously decreasing rotational speed of the shaft was also applied in the form of an amplitude curve at the same surface.

### 2.5. Analysis Step

The step for the entire shutdown process was set to 2 s and the number of sampling points in the field output was set to 20,000 such that the sampling frequency was 5000 Hz. Each sampling point was output at exact times and evenly spaced time intervals. The maximum number of increments was set to 25,000 to avoid computation failures due to a lower convergence speed. The position of the observation point (N502) is shown in Figure 3, and the vibration data of this point in the x-direction was adopted as a criterion for the bearing vibration in the later analysis.

## 3. Results and Discussion

There are many factors that have a certain effect on the vibration of a bearing system. This part will use the transient dynamics method in finite element analysis (FEA) to study the effects of four factors on friction characteristics and vibration noise for a WLRB system during a shutdown process. Mastering the effects of the friction coefficient, specific pressure, temperature, and stiffness of a bearing system on the vibration characteristics and friction noise of a WLRB could provide a theoretical reference for reducing vibration during a shutdown process.

### 3.1. Effects from the Friction Coefficient

**Figure 4:**
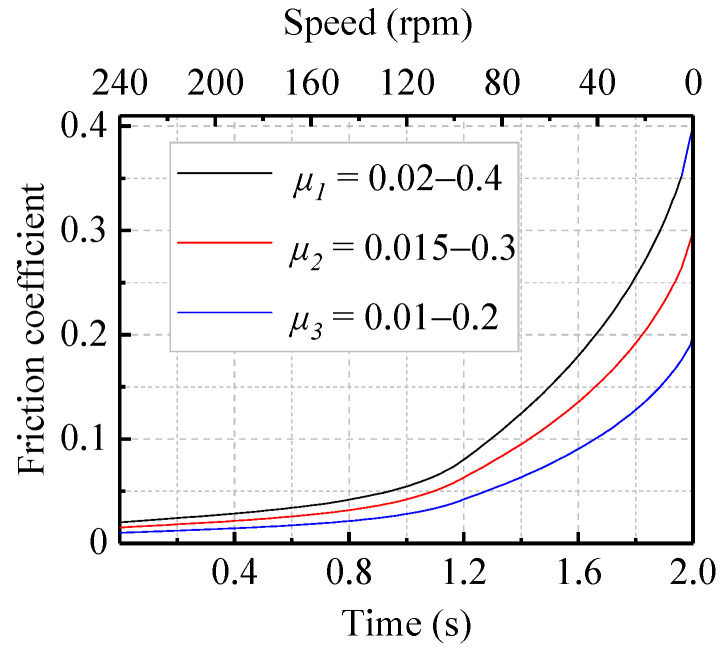
The curves relating the time, speed, and friction coefficient.

Friction is the most immediate factor that induces vibration, while the vibration and noise from a WLRB are also caused by friction; therefore, the friction coefficient is the factor that is most worth studying in the analysis of vibration and noise. Usually, the friction coefficient is considered to be a constant, which is the ratio of the frictional force to the normal pressure. However, a large number of experiments have shown that the friction coefficient between a shaft and a bearing fluctuates and it is irregular during operation. Especially under the condition of low speed and a heavy load, boundary lubrication or even a dry friction state appears on the contact surface, and the noise generated under this condition is closely related to friction. In view of this, by setting the three friction coefficient curves during the same shutdown process (Figure 4) according to a U.S. military specification (MIL-DTL-17901C (SH) [26]) and the related research [27], this study investigated the effects of different friction coefficients on vibration characteristics and frictional noise for a bearing system. At a specific pressure of 0.4 MPa, a temperature of 20 °C, and a speed that was linearly changing from 240 to 0 rpm, the friction coefficient for the three cases was 0.01–0.2, 0.015–0.3, and 0.02–0.4, respectively.

**Figure 5:**
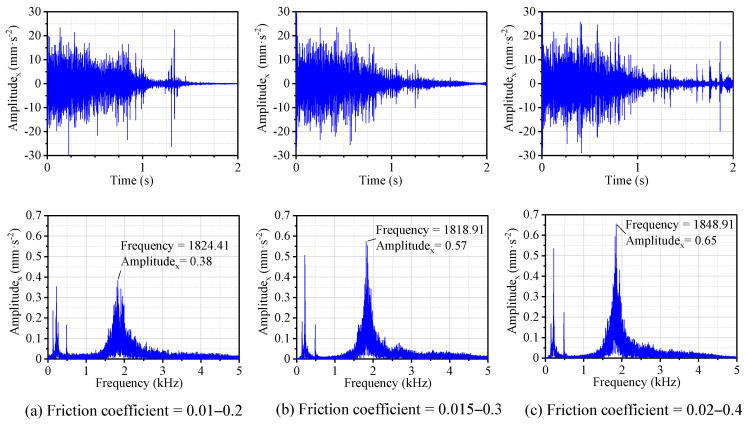
Displacement and frequency spectrum of N-502 with different friction coefficients.

**Figure 6:**
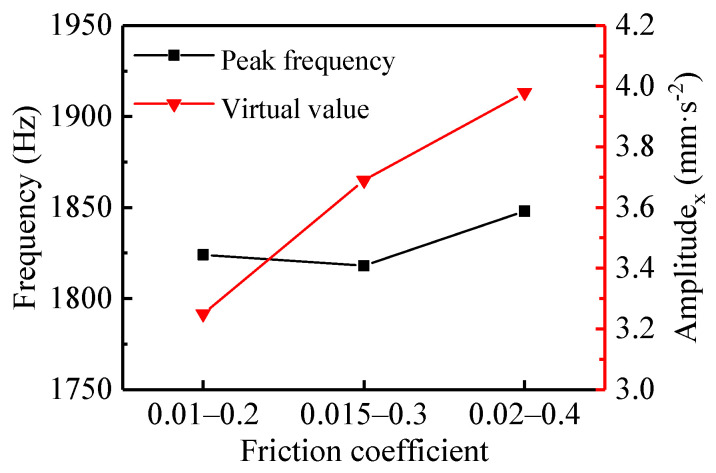
The peak frequency and root mean square (RMS) in the x-direction with different friction coefficients.

Figure 5a–c display the time and frequency domain responses of the vibration acceleration of N-502 in the x-direction corresponding to the friction coefficient from 0.01–0.2, 0.015–0.3, and 0.02–0.4, respectively. The acceleration signal in the time domain mainly characterizes the energy strength of the vibration, while the frequency domain signal represents the distribution of the frequency band of the vibration. The non-symmetric load was set to 1280 N and the rotational speed of the shaft was linearly reduced from 240 to 0 rpm. Based on the acceleration signal in the time domain, the virtual values (root-mean-square values) of the vibration acceleration of N-502 were calculated, as shown in Figure 6.

From the time domain results in Figure 5a–c, the displacement of the vibration acceleration from 0 to 0.5 s was roughly increasing and the trend became obvious with the increase of the friction coefficient, while the displacement from 0.5 to 2 s was decreasing, which agrees with the shared understanding that stick-slip can occur when the friction coefficient decreases with increasing speed [28,29]. Combining Figure 5 and Figure 6, with the increase of the friction coefficient during the shutdown process, it can be seen that the peak frequency corresponding to the friction coefficients of 0.01–0.2, 0.015–0.3, and 0.02–0.4 were 1824, 1819, and 1849 Hz, respectively; the virtual value of the vibration acceleration increased from 3.25 to 3.98 mm·s^−2^, which is consistent with practice and experience stating that noise with a higher decibel level could be generated with the increasing friction coefficient. Overall, the virtual value of acceleration increased obviously as the friction coefficient increased; the peak frequency also did, but not obviously.

### 3.2. Effects from the Specific Pressure

The contact area between the shaft and the bearings is another factor that affects the vibration state of a bearing system, and the load directly determines the contact area. As the load increases, the effective contact area also increases. Regarding this connection, this section applied the unequal-sized load under the same friction coefficient curve and deceleration process to obtain results both in the time and frequency domains. Finally, the effects of different specific pressures on the vibration characteristics during the same shutdown process were discussed. Three sets of data were utilized in this study, and the friction coefficient–speed curve during the shutdown process was the *μ*_1_ shown in Figure 4. The specific pressures for the three sets of data were, respectively, 0.4, 0.5, and 0.6 MPa with the *μ*_1_ friction coefficient (Figure 4), the speed going from 240 to 0 rpm, and a temperature of 20 °C.

**Figure 7:**
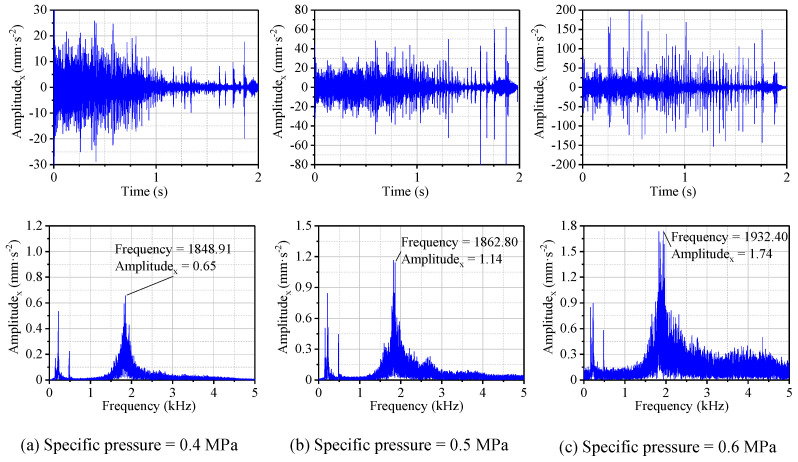
Displacement and frequency spectrum of N-502 under different specific pressures with the *μ*_1_ friction coefficient.

**Figure 8:**
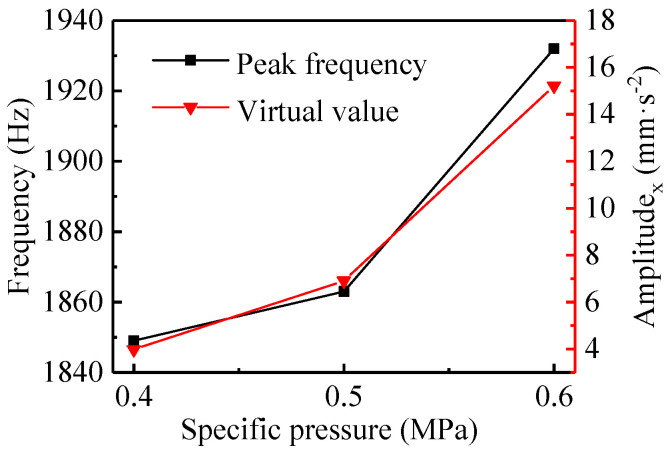
The peak frequency and RMS in the x-direction under different specific pressures with the *μ*_1_ friction coefficient.

Figure 7 shows the results in the time and frequency domains under loads of 1280, 1600, and 1920 N, i.e., the specific pressures of 0.4, 0.5, and 0.6 MPa, respectively. Based on the time domain data in the shutdown process, the virtual values of the vibration acceleration for the bearing system under the three specific pressures above were calculated, as shown in Figure 8.

As the load increased, the time domain vibration was more complex and the vibration amplitude became greater. The increase in the specific pressure expanded the speed range where the friction-induced vibration occurred. Combining Figure 7 and Figure 8, it can be seen that the virtual value of the vibration acceleration increased with the increase of the specific pressure throughout the shutdown process, and the virtual values of the acceleration for the specific pressures of 0.4, 0.5, and 0.6 MPa were 3.98, 6.91, and 15.21 mm·s^−2^, respectively. In addition, the results of the frequency domain reveal that as the specific pressure increased, the frequency corresponding to the vibration peak increased in the order of 1849, 1863, and 1932 Hz. The reason was that increasing the load resulted in greater contact pressure and frictional force [30,31] between the shaft and the bearing. Rubber is a hyperelastic material and greater contact deformation is more likely to cause greater bearing vibration. As a consequence, the greater the load is, the more likely a WLRB is to generate vibration noise with a high decibel level and squeal. In addition, if the specific pressure is set to 0.7 MPa or greater, it may have a more prominent effect on the friction characteristics and vibration noise of the bearing system.

### 3.3. Effects from Temperature

The reason why sliding bearings have their own unique properties compared with rolling bearings is that an oil film has a strong capacity to absorb and damp vibration, which makes operations stable and reliable. Under the actual operating conditions of a WLRB, with an increase in the load, the bearing bush hardly continues to operate in the absence of lubricants and coolants supplement, resulting in heat generation and dispersion in the friction belt. Overheating generated in the friction belt usually causes the rubber bush to undergo a rapid adhesive wear process, where the sliding bearing bush is burned out and even a journal sticking phenomena appears [12]. Thermal expansion and deformation of rubber are so considerable compared to the small radial clearance that the bush contacts the shaft, the radial clearance decreases, and working resistance increases, finally resulting in bearing failure, especially in the case of silt and other impurities being present in the water. Friction determines whether overheating occurs in the friction belt, and usually, overheating acts on friction in turn. Therefore, a water-lubricated bearing bush cannot withstand too much heat, and most of the heat generated in a friction belt must be absorbed by the flowing lubricants. It is also necessary to set the temperature as monitoring information for a WRLB under certain operating conditions. However, the working performance at a high temperature is of great importance for a WRLB; therefore, the development of new high-temperature-resistant bush materials has attracted attention from researchers. In this study, the effects of temperature on vibration noise were investigated by setting different temperatures on the rubber surface that was in contact with the shaft during the shutdown process. The temperatures for the three sets of data were 30, 35, and 40 °C with the *μ*_1_ friction coefficient, the speed going from 240 to 0 rpm, and a specific pressure of 0.6 MPa.

**Figure 9:**
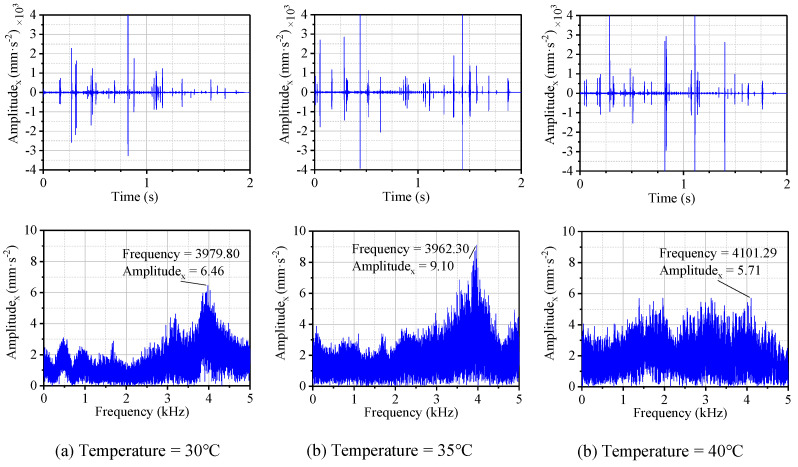
Displacement and frequency spectrum of N-502 under different temperatures with the *μ*_1_ friction coefficient.

**Figure 10:**
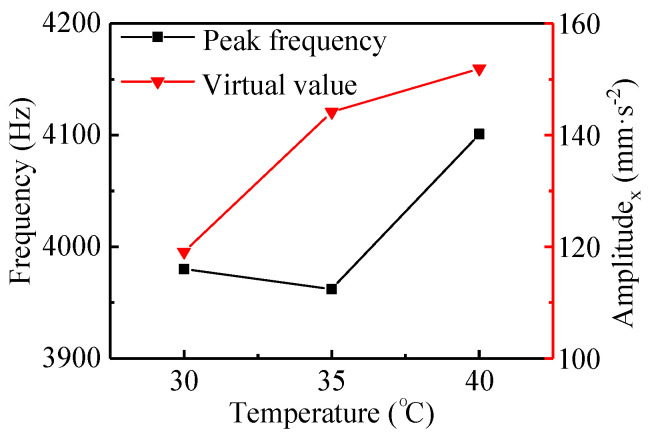
The peak frequency and RMS in the x-direction under different temperatures with the *μ*_1_ friction coefficient.

Figure 9a–c shows the results in the time and frequency domains with the temperatures of 30, 35, and 40 °C, respectively. Based on the time domain results in the shutdown process, the virtual values of the vibration acceleration for the bearing system under the three temperatures mentioned above were calculated, as shown in Figure 10.

As can be obtained from Figure 7c and Figure 9a, under the same conditions, the vibration amplitude of the bearing with a temperature of 30 °C was significantly higher than that with a temperature of 20 °C, indicating that the rubber material was significantly sensitive to the increase in temperature. The reason for this was that with the increase of temperature, the activity of the rubber surface increased, the hardness decreased, and the deformation increased, resulting in increased viscosity and increased frictional vibration [9]. It makes sense to increase the rubber’s thermal resistance by modifying the rubber or polymer materials.

### 3.4. Effect from the Stiffness of the Bearing Support

There are two basic measures of controlling structural vibration, namely, active vibration attenuation and vibration isolation. Vibration isolation refers to the installation of a pad between the vibration source and the base, which relies on the deformation of the pad to buffer the effect of the vibration source’s excitation on the structure, absorb vibration energy transmitted from the vibration source, and adjust the stress state of structure to increase the compression area between the structure and the pad. In addition, this method has little impact on ship equipment and is a relatively small-scale project, especially for the improvement of the ships that are designed or even built. The vibration isolator consists of an elastic element that acts as a supporting role and a damping element; therefore, it has a certain stiffness and damping. Rubber, a metal spring, cork, fiberglass, and an air cushion are some common vibration isolation materials that can be used alone or in a combination to meet different requirements for vibration isolation.

With the scale of modern ships getting larger, the power output of the spindle and propeller increase and the shafting components become larger accordingly. The vibration isolation equipment is used on the vibratory bearing to weaken the vibration and decrease the vibration transmission efficiency. When a bearing system is running, the friction-induced vibration and shock among the components increase the working load of a marine propulsion system; therefore, it is of great importance to install adequate vibration isolation measures for marine machinery and equipment. As far as a marine stern bearing is concerned, this will lead to a certain amount of bending deformation due to the cantilever action of gravity on the propeller, resulting in an inadequate contact area, uneven contact pressure, and excess local pressure between the propulsion shaft and the stern bearing. In this way, one end of a marine stern bearing is subjected to a larger pressure, resulting in deformation and a block of water grooves. Finally, poor lubrication and fluctuation in the friction coefficient cause uneven wear of the bearing bush and vibration of the bearing system. The whole process described above is called the “edge effect.” The stiffness of the bearing support generates eccentricity and misalignment in the bearing system, which can also lead to edge effects. In this study, the effect of the stiffness of the bearing support on the bearing vibration was studied by connecting the spring and damper to the ground. Stiffness values of 2 × 10^6^, 2 × 10^11^, and 2 × 10^13^ N·mm^−1^ were set for the three cases. A specific pressure of 0.4 MPa, a temperature of 20 °C, the *μ*_3_ friction coefficient, and a damping factor of 21,120 kg·s^−1^ were selected for the three cases.

**Figure 11:**
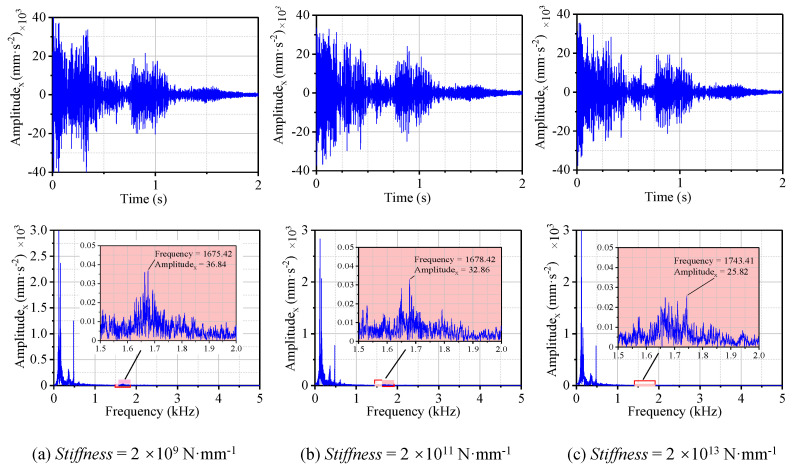
Vibration acceleration displacement and frequency spectrum of N-502 under different stiffness values of bearing support with the *μ*_3_ friction coefficient.

**Figure 12:**
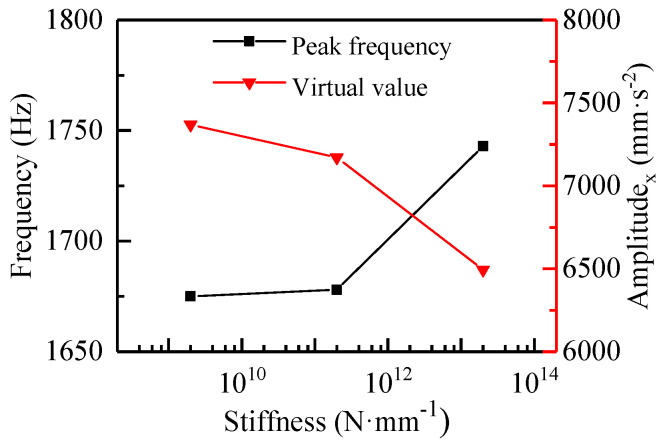
The peak frequency and RMS in x-direction under different stiffness values with the *μ*_3_ friction coefficient.

The time and frequency domain results for the three cases above are shown in Figure 11, and the virtual values of the acceleration were calculated, as shown in Figure 12. With the increase of the spring stiffness between the bearing and bearing support, the amplitude of the vibration acceleration in the high-frequency band was greatly reduced compared with that in the low-frequency band (Figure 11). The acceleration peak for the stiffnesses of 2 × 10^9^, 2 × 10^11^, and 2 × 10^13^ N·mm^−1^, and the fully fixed bearing (Figure 5a) were 36.84, 32.86, 25.82, and 0.38 mm·s^−2^, respectively. Compared with the fully fixed bearing, the peak frequency in the high-frequency band decreased with the decrease of the spring stiffness. For the stiffness of the fully fixed bearing and the stiffnesses of 2 × 10^13^, 2 × 10^11^, and 2 × 10^9^ N·mm^−1^, the corresponding frequencies were 1824, 1743, 1678, and 1675 Hz; that is to say, the lower the stiffness was, the lower the peak frequency. However, the acceleration peak in the low-frequency band was greatly increased to more than 3 × 10^3^ mm·s^−2^, resulting in particularly large virtual values of acceleration compared with the fully fixed situation. As the stiffness of the bearing support increased, the virtual value of the acceleration decreased in the order of 7369.56, 7171.55, and 6493.91 mm·s^−2^ (Figure 12). The reason for the results above was that the application of a partial load gave rise to a certain degree of misalignment, and now a serious misalignment between the bearing and the shaft was generated because of the introduction of the spring. Both theory and practice show that misalignment and eccentricity between shafts and bearings are the main causes of vibration [32]. Therefore, it is of great value to choose a combination of appropriate stiffness and damping that can get a smaller vibration as far as possible.

## 4. Orthogonal Design

In multi-factor and multi-level trials, if each level of each factor is matched to each other for a comprehensive experiment, there will be a lot of trials that need to be conducted. For the vibration of a WLRB, four factors—friction coefficient, specific pressure, temperature, and stiffness of bearing support—were considered in this study, each of which contained three levels (Table 2). In order to study the degree of influence of each factor on the vibration, each level of each factor was properly combined to obtain the minimum peak frequency and virtual value. For four factors with three levels each, if each level of four factors is paired with each other, this requires 3^4^ times trials. The purpose of an orthogonal design is to minimize the number of trials without affecting the effect of the trial. In this study, an orthogonal table (L_9_(3^4^)) was designed to study the effects of these four factors on the two indicators (peak frequency and virtual values of acceleration (Table 3)).

As can be seen from Table 3, the range of the specific pressure, whether for peak frequency (727.30 Hz) or virtual value acceleration (3292.01 mm·s^−2^), was large; that is to say, the specific pressure was a factor that had a great influence on the two indicators. Although the range of stiffness (7205.94 mm·s^−2^) was the largest for the virtual value of acceleration, this was caused by a serious misalignment because of a decrease in stiffness. After the increase of pressure, the rubber’s stick-slip was prominent, and then, the rubber deformation was greater. The vibration caused by the recovery of the elastic deformation was also increased. Considering these two indicators together, the low load, i.e., level 1, was the best.

As shown in Table 3, the range for stiffness was large in terms of the peak frequency and the virtual value of acceleration, i.e., the stiffness was an influential factor. It is clear that level 2 or 3 was the best choice in terms of reducing the peak frequency, but level 1 was the best in terms of reducing the virtual value. However, from the previous analysis, it can be concluded that the reason for the sharp increase (from 697.32 to 22,315.13 mm·s^−2^) in the virtual value of the acceleration was due to the serious misalignment caused by the decrease in the stiffness, which can be corrected by precise assembly or by modifying the rubber materials to enhance the vibration absorption performance and resistance to misalignment. Therefore, level 3 was the best choice.

Table 3 shows that the range of the friction coefficient was not the biggest, whether for the peak frequency (717.13 Hz) or the virtual value (1184.58 mm·s^−2^), and the increase in the friction coefficient had the opposite effect on the peak frequency and the virtual value. Level 1 (4978.75 Hz) was the best in terms of reducing the peak frequency, and the results of the previous analysis for the friction coefficient revealed that level 1 was also the best, while the reason that opposite results for the virtual value (from 5076.28 to 3891.69 mm·s^−2^) were found was mainly due to the impact of the specific pressure and the stiffness. It can be seen that trial of 3 consisted of the load, temperature, and stiffness at level 3, which made the sum of the virtual value (15,228.84 mm·s^−2^) of trials of 1, 2, and 3 the largest, while the friction coefficient did not contribute to the maximum value much. Similarly, the sum of the virtual value of the acceleration (13,508.04 mm·s^−2^) at level 2 for the friction coefficient was also the combination of a large specific pressure (level 3) and a low stiffness (level 2). The sum of the virtual value of acceleration at level 3 (11,675.08 mm·s^−2^) for the friction coefficient was the smallest because of the combination of a high friction coefficient (level 3) and the maximum stiffness (level 1). Therefore, level 1 was the best choice for the friction coefficient.

The sum of the peak frequency (5006.25 Hz) at level 1 for temperature was the smallest, while for the virtual value of the acceleration, level 2 (10,740.01 mm·s^−2^) was the smallest. The reason for this was that the contribution of the temperature at level 1 to the sum of the virtual value was small, while the specific pressure and stiffness were the main factors. It can be seen that the specific pressure and stiffness of trials of 6 and 8 were set at levels 3 and 2 and levels 2 and 3, respectively, which significantly contributed to the increase in virtual values. Therefore, level 1 was the best choice for temperature.

Through the comprehensive analysis for each factor and its levels, a better test scheme was obtained:

- B1—specific pressure, level 1, 0.3 MPa.
- D3—stiffness of bearing support, level 3, 2 × 10^9^ N·mm^−1^.
- C1—temperature, level 1, 20 °C.
- A1—friction coefficient, level 1, 0.01–0.2.

In addition, the results of trials of 1, 5, and 9 show that only under the action of a large pressure could the temperature cause the vibration noise with a high peak frequency, namely squeal.

## 5. Conclusions

In this study, a finite element model of a WLRB system was established to study its vibration, where the shutdown process was simulated by setting a friction coefficient curve. The effects of the friction coefficient, specific pressure, temperature, and the stiffness of the bearing support on the vibration were discussed. An orthogonal table (L_9_(3^4^)) was also used to study the degree of influence of four factors on the vibration characteristics of a WLRB. The following results were found:

(1) The friction coefficient and specific pressure played a similar role in causing the vibration of a WLRB; however, obviously, increased specific pressure is more likely to lead to an increase in the virtual value and peak frequency of the acceleration compared with an increased friction coefficient.

(2) The carrying capacity of the bearings under high-temperature conditions was lower than that of bearings under low-temperature conditions. Decreasing the spring stiffness of the bearing support could reduce squeal; however, at the same time, it increased the noise level in the low-frequency band.

(3) The finding that a higher squeal can be caused by a higher temperature was due to the presence of large specific pressure.

## Figures and Tables

**Table 1:** Bearing parameters.

Parameters	Values
External diameter, D (mm)	60
Inner radius, d (mm)	40
Length, L (mm)	80
Radius of the groove, r (mm)	3
Groove number, n	8
Rubber thickness, h (mm)	6
Copper thickness, B (mm)	4

**Table 2:** Parameter settings.

Level	A Friction Coefficient	B Pressure (MPa)	C Temperature (°C)	D Stiffness of Bearing Support (N·mm^−1^)
1	0.01–0.2	0.3	20	Fixed
2	0.015–0.3	0.4	25	2 × 10^9^
3	0.02–0.4	0.5	30	2 × 10^11^

**Table 3:** Orthogonal table.

	Factor	1	2	3	4	Results	
No.		A	B	C	D	Frequency (Hz)	RMS (mm·s^−2^)
1		1	1	1	1	1629.42	10.06
2		1	2	2	2	1662.42	4847.95
3		1	3	3	3	1686.92	10,370.83
4		2	1	2	3	1654.92	5208.48
5		2	2	3	1	1653.97	3.69
6		2	3	1	2	1684.92	8295.86
7		3	1	3	2	1671.92	4255.69
8		3	2	1	3	1691.92	6735.82
9		3	3	2	1	3766.31	683.58
Frequency (Hz)	K_1_	4978.75	4956.25	5006.25	7049.70		
	K_2_	4993.80	5008.30	7083.65	5019.25		
	K_3_	7130.14	7138.14	5012.80	5033.75		
	κ_1_(K_1_/3)	1659.58	1652.08	1668.75	2349.90		
	κ_2_(K_2_/3)	1664.60	1669.43	2361.22	1673.08		
	κ_3_(K_3_/3)	2376.71	2379.38	1670.93	1677.92		
	Range	717.13	727.30	692.47	676.82		
	Optimum solution	A1	B1	C1	D2		
RMS (mm·s^−2^)	K_1_	15,228.84	9474.23	15,041.74	697.32		
	K_2_	13,508.04	11,587.46	10,740.01	17,399.50		
	K_3_	11,675.08	19,350.27	14,630.21	22,315.13		
	κ_1_(K_1_/3)	5076.28	3158.08	5013.91	232.44		
	κ_2_(K_2_/3)	4502.68	3862.49	3580.00	5799.83		
	κ_3_(K_3_/3)	3891.69	6450.09	4876.74	7438.38		
	Range	1184.58	3292.01	1433.91	7205.94		
	Optimum solution	A3	B1	C2	D1		
